# Teacher-assigned grades and external exams: sources of discrepancy

**DOI:** 10.1080/0969594X.2024.2338764

**Published:** 2024-04-11

**Authors:** José Manuel Arencibia Alemán, Astrid Marie Jorde Sandsør, Henrik Daae Zachrisson, Sigrid Blömeke

**Affiliations:** a Centre for Educational Measurement, University of Oslo, Oslo, Norway; b Department of Special Needs Education, University of Oslo, Oslo, Norway

**Keywords:** Teacher-assigned grades, external exams, concurrent validity, Norway

## Abstract

Modest correlations between teacher-assigned grades and external assessments of academic achievement (*r* = .40–.60) have led many educational stakeholders to deem grades subjective and unreliable. However, theoretical and methodological challenges, such as construct misalignment, data unavailability and sample unrepresentativeness, limit the generalisability of previous findings. We overcome these challenges by exploiting rich, population-wide data from the National Registries in Norway (*n* = 511,858), where state regulations require close construct alignment between grades and external exams. Correlations between lower-secondary education final grades and external exam results (*r* = .64–.86) suggest that grades are better measures of academic achievement than previously acknowledged. Dominance analyses and multivariate regression analyses indicate that external exam results are the best predictor of grades in the same subject. However, our results also indicate that state regulations and quality assurance systems cannot completely eradicate potential sources of discrepancy.

## Introduction

In many countries, teacher-assigned grades (hereafter *grades*) are among the most consequential high-stakes academic achievement measures that secondary education students receive and thus, the valid use and interpretation of grades is crucial (American Educational Research Association AERA, American Psychological Association APA, & National Council on Measurement and Education NCME, 2014). However, previous studies suggest that grading practices differ across subjects (Prøitz, 2013); that teachers’ abilities to evaluate students’ academic achievements may depend on those students’ characteristics, such as conscientiousness (Westphal et al., 2021); and that school culture may determine the extent to which teachers consider non-achievement elements while grading (Lekholm & Cliffordson, 2008). Are grades reliable, accurate measures of academic achievement? We address this validity issue by examining grade variance, asking how much is explained by quasi-concurrent external exams of the same target construct and how much is explained by potential sources of discrepancy (Willingham et al., 2002).

The underlying assumption in concurrent validation studies is that grades and external assessments should be unbiased measures of the same academic achievement construct. Thus, highly correlated grades and external exams would provide concurrent validity evidence for the interpretation of grades as measures of academic achievement. However, most concurrent validation studies stem from the USA, and they often focus on the association between grade point average (GPA) and test scores (Brookhart et al., 2016, p. 810). Since neither the content taught nor the content assessed by grades or standardised tests in the USA is guided by a national curriculum (U.S. Department of Education, 2008), validity arguments that rely on criterion-related evidence may be inappropriate (Brookhart et al., 2016). Furthermore, GPA often encompasses different subjects and thus may fail as an indicator of comparable academic achievement (Wittman, 2022).

Studies focused on same-subject measures, particularly from outside the USA, are much sparser. The few available studies of the association between same-subject grades and external assessments point to a shared variance between 16% and 36% (Ross & Kostuch, 2011). However, these findings are inconclusive due to small sample sizes, limited data availability and limited construct overlap (Brennan et al., 2001; Meissel et al., 2017; Ross & Kostuch, 2011). Furthermore, the contexts of some studies in this strand of the literature pose additional challenges. For example, in Sweden, teachers use national tests to calibrate their grades (Lekholm & Cliffordson, 2009) and, in Spain, discrepancies between grades and low-stakes external assessments might be driven by differences in student motivation (Gortazar et al., 2022). Thus, the questions of how much grade variance is explained by academic achievement and what elements other than academic achievement are measured by grades remain partially unanswered.

Our study contributes to the literature by examining the association between independent and quasi-concurrent internal and external academic achievement measures – Year-10 grades and national written exams in Norway – legally required to measure the same academic achievement construct – curriculum-based competence goals – which have the same weight in students’ GPAs. Thus, ‘a validity argument that expects grades and [external exams] to correlate highly’ is appropriate (Brookhart et al., 2016, p. 824). Rich, population-wide data from the Norwegian National Registries for all Year-10 students in Norway between 2010 and 2018 (*N* = 574,096), allow us to overcome previous methodological limitations. Our results may be useful for stakeholders interested in the potential and limitations of requiring grades and external exams to measure the same curriculum-based competence goals.

## Theoretical background

### Teacher-assigned grades and external assessments

Typically, grades refer to teachers’ summative evaluations of student academic achievement. However, what academic achievement is and how teachers collect and aggregate evidence of academic achievement vary across educational systems, and schools, subjects, teachers and students. In contrast, external assessments of academic achievement are typically developed, administered and assessed in some standardised way by stakeholders other than students’ teachers.

In the USA and many other Western countries, grades have predominantly evolved from norm-referenced measures of an aspect of intelligence to individual-referenced measures of learning progress and then to criterion-referenced measures of academic achievement (Brookhart, 2015). Methodological advances and renewed theoretical foundations have motivated research suggesting that grades are multidimensional measures of what teachers value in student work (Brookhart et al., 2016). For example, in Sweden, Lekholm and Cliffordson (2008) identified subject-specific academic achievement dimensions loading on grades and national test results in the same subject and a common dimension loading on all grades capturing non-cognitive factors valued by teachers. Similarly, Bowers (2011) suggested the existence of a ‘non-cognitive classroom behaviour dimension’ underlying the discrepancy between grades and test scores, and an ‘academic knowledge dimension’ (p. 151) underlying the discrepancy between assessments in core and non-core subjects in the US.

Thus, controversies surrounding the validity and fairness of academic achievement measures based on the assumption that external assessments and grades are mutual surrogates might be unjustified (Willingham et al., 2002).

### Sources of discrepancy

We build on Willingham et al. (2002, p. 4) ‘Framework of Possible Sources of Discrepancy Between Observed Grades and Test Scores’ (hereafter, *Willingham’s framework*). In this framework, discrepancies in the rank order of students according to grades and external assessments can be explained by content differences, individual differences, situational differences and measurement errors.

#### Content differences

Willingham’s framework considers three types of content differences: differences in the knowledge domain assessed, e.g. between vocabulary and grammar in English assessments; differences in the skills required to perform across assessments, e.g. between oral and written assessments; and differences in elements other than knowledge domain and skills that may influence grades or external assessments, e.g. student effort or test wiseness (Willingham et al., 2002).

State regulations in Norway require grades and external exams to measure the same construct. Thus, systematic differences in the knowledge domain or non-achievement factors assessed by grades and external exams should not exist. However, while teachers are required to collect varied evidence of academic achievement, external written exam results are based on limited evidence collected within the constrained formats and durations of exams. Thus, there may exist differences in the skills required to perform across different assessments that, if not accounted for, would result in ‘unduly conservative’ (Willingham et al., 2002, p. 1) estimates of concurrent validity evidence. For example, some studies have suggested that reading comprehension is a better predictor of written expression than oral expression (Berninger & Abbott, 2010). If the association between reading skills and performance in written assessments was positive, poor readers might be better able to demonstrate academic achievement in oral assessments – which integrate the portfolio of evidences collected by teachers – than written assessments – such as written exams. Thus, controlling for national reading test scores may help to improve the concurrent prediction of grades by external exams.

#### Individual differences

Individual differences concern the degree to which students’ learning experiences outside school, student characteristics that may affect academic motivation and teachers’ judgements of student academic achievement interact with content differences (Willingham et al., 2002). For example, if foreign background was negatively associated with reading skills (Marx & Stanat, 2012), their interaction might lead to larger discrepancies between the grades and written exam results of students with foreign backgrounds (Berninger & Abbott, 2010). In such specification, the non-interacted associations between student characteristics and grades might be better indicators of content differences related to non-achievement factors – i.e. grading practises that threaten grade validity.

Previous research suggests that teachers favour girls when assigning grades (Bonesrønning, 2008), that socioeconomic status might be associated with learning experiences outside school that contribute to better performance on external assessments (Willingham et al., 2002) and that family structures might impact teachers’ judgements of student academic achievement through, for example, students’ emotional status (Keller, 2016; Westphal et al., 2021). Thus, student characteristics such as gender, parental education, family income and structure or national background might be good indicators of individual differences.

#### Situational differences

Across cohorts and institutional contexts, within-individual variation – e.g. learning progress and motivation – and variation in the content, context and format of assessments may explain observable discrepancies between grades and external assessments (Willingham et al., 2002).

In Norway, all Year-10 students take their written exams simultaneously at their local schools. Thus, content, context and format discrepancy should be cohort- and school-dependent. Moreover, grade assignment and written exam administration are quasi-concurrent. Thus, within-individual variation should be negligible.

#### Measurement error

Systematic errors at the student level – e.g. resulting from cheating – the teacher level – e.g. grading leniency – and the school level – e.g. school-related differences in grading standards – may explain observed discrepancies between grades and external exams. Moreover, unsystematic errors or unreliability would attenuate their correlation (Willingham et al., 2002).

Accounting for measurement errors based on observed final grades and exam results is impossible. However, indicators of individual and situational differences would control part of the variation due to systematic errors associated with student and school characteristics. Furthermore, estimates of inter-rater reliability in upper-secondary external exams (Björnsson & Skar, 2021) might provide a fair benchmark to study the influence of unreliability on concurrent validity estimates.

## Current study

We examine the extent to which academic achievement – as measured by external written exams – and potential sources of discrepancy explain the variance of Year-10 grades in ‘Mathematics’ (*mathematics*), ‘Norwegian, first-choice form of Norwegian, written’ (*Norwegian*)^1^ and ‘English, written’ (*English*). We circumvent concerns about the appropriateness of criterion-related evidence in building validity arguments by focusing on the Norwegian educational assessment system. Rich, longitudinal, high-quality administrative data from the Norwegian National Registries allow us to also reduce the risks associated with issues such as time bias, sample bias or model misspecification.

### Research questions and hypotheses

This study addresses the following research questions:

1. How much of the variance in mathematics, Norwegian and English grades is explained by construct-equivalent written exams in Norway?
2. How much of the variance in mathematics, Norwegian and English grades is explained by indicators of content differences, individual differences, situational differences and measurement errors in Norway?
3. Does the variance in grades explained by written exams and possible sources of discrepancy differ across subjects in Norway?

Three hypotheses underlie our research questions. First, since state regulations require grades and external exams in Norway to measure the same construct, their association should be stronger than previously suggested, i.e. *r* > .60 (Ross & Kostuch, 2011). Second, despite state regulations and quality assurance measures intended to increase the inter-rater reliability and validity of written exam results (Directorate for Education and Training DET, 2017; Regulation of the Education Act, 2006), sources of discrepancy may have independent contributions to the explained variance in grades. Finally, previous studies suggest that mathematics teachers use standardised grading tools and that mathematics and Norwegian teachers rely heavily on previous written exams to collect evidence of academic achievement (Prøitz, 2013). Using standardised grading tools may increase the reliability of mathematics grades. Relying on previous exams has the potential to enhance format consistency between evidence of academic achievement collected by teachers and in external exams in mathematics and Norwegian. If so, we should find stronger estimates of concurrent validity in mathematics than in Norwegian and English, respectively.

## The Norwegian context

### Teacher-assigned grades in Norway

Generally, children in Norway start their comprehensive compulsory education the year they turn six (Education Act, 1998), which encompasses primary education (Years 1–7) and lower-secondary education (Years 8–10). Grade retention and grade skipping during compulsory education in Norway are rare, so most students graduate from lower-secondary education the year they turn 16.

From Year 1 onward, students receive unstandardised, non-numeric evaluations of curriculum-based academic achievement at the end of each term (*halvårsvurdering*). These biannual end-of-term evaluations satisfy summative and formative purposes, providing students and their parents with information about academic progression and guidance on how to further develop the curriculum-based competences that define academic achievement in Norway.

In Year 8, students start receiving criterion-referenced, low-stakes, numeric grades that supplement end-of-term subject evaluations. These grades report curriculum-based competence on a 6-point scale with values of 1 (very low), 2 (low), 3 (quite good), 4 (good), 5 (very good) or 6 (excellent). The final numeric grades that students receive in the last term of Year-10 (*standpunktkarakterer*) are part of the GPA used in the admissions process to upper-secondary education programmes – which is calculated as the arithmetic average of grades and external exam results multiplied by 10 – and, therefore, high-stakes academic achievement measures (DET, 2021). Thus, the regulatory framework implicitly requires grades – and external exam results – to satisfy interval properties – e.g. a grade 4 should contribute twice as much as a grade 2 to student GPA.

In some cases, students may receive non-numeric grades. For example, teachers who cannot collect enough evidence to assign numeric grades due to student absenteeism assign an IV (*ikke vurderingsgrunnlag*, ‘without grading basis’), and students with special education needs exempted from numeric grades receive an F (*frittat*, ‘exempt’). IV grades without valid documented reasons – e.g. medical permit – contribute with 0 points to GPA calculations. Other non-numeric grades are excluded from GPA calculations.

In 2008, the Norwegian national curriculum strengthened the conceptualisation of grades as criterion-referenced measures by outlining the competences students should acquire at different levels (Tveit, 2014). It also required teachers to provide multiple, varied opportunities for students to demonstrate their curriculum-based competence, potentially increasing the reliability and validity of grades (Smith, 2003). Nevertheless, based on 41 semi-structured interviews with secondary education teachers in Norway, Prøitz (2013) suggested that mathematics and Norwegian teachers base their grades mostly on written assignments and that teachers were more lenient towards participative students with lower levels of curriculum-based competence. Thus, construct underrepresentation, construct-irrelevant elements or both may affect grades’ validity.

### Written exams in Norway

In addition to final grades, Year-10 students receive one locally administered semi-external oral exam result (see ‘Oral exams in Norway’) and one nationally administered external written exam result.

Whether students take their written exam in mathematics, Norwegian^2^ or English is randomly determined through an ‘exam lottery system’ (*trekkordning*). Like grades, written exams report curriculum-based competence on a 6-point ordinal scale or, occasionally, non-numeric values – e.g. IM (*ikke møtt*, ‘not met’) if the student does not take the exam to which they were assigned. However, the regulatory framework implicitly acknowledges that demonstrations of academic achievement during exams may deviate from actual curriculum-based competence when it clarifies that exams measure curriculum-based competences as *demonstratedduring exams* (Regulation of the Education Act, 2006, § 3–22).

There are several quality assurance measures to ensure the reliability and validity of written exams (DET, 2017). For example, to minimise differential item functioning, the DET selects written exam developers (*fagnemndene*) from among teachers from different parts of the country. The goal is to guarantee that the nationally diverse backgrounds of students are considered while developing written exam tasks. Furthermore, exam developers must ensure that written exam tasks allow students at all levels to demonstrate their curriculum-based competence.

The DET selects raters (*sensorer*) from among practising teachers recommended by school principals. These raters are invited to participate in rater training seminars (*sensorskolering*) focused on the use of nationally homogeneous criteria. During the rating process, raters first evaluate exam tasks independently, then discuss disagreement and finally agree on a common result. The DET also selects exam coordinators (*oppmennene*) from among teachers to assist in rater training and, during the assessment process, as third and decisive raters if exam raters are unable to agree on a result (DET, 2017).

In conjunction, these and other quality assurance routines should ensure the reliability and validity of written exam results. Nevertheless, Björnsson and Skar (2021) found worrying degrees of initial examiner disagreement in upper-secondary education. Preliminary rater evaluations had inter-rater intra-class correlations between .91 and .93 in mathematics exams, but only between .62 and .64 in Norwegian exams and .68 in English exams. Since the structure and organisation of lower- and upper-secondary education exams are nearly identical, an equivalent analysis of lower-secondary exams would likely show similar results.

### Oral exams in Norway

Year-10 students are also randomised to one local oral exam in mathematics, English, Norwegian, the natural sciences, the social sciences, religion or a third, elective foreign language. In addition to format and duration – oral versus written, and 30 minutes instead of 5 hours – two important differences distinguish oral from written exams. First, the regulatory framework does not require both examiners to be external during oral exams, and, in practice, teachers usually assess their own students (Tveit, 2014). Moreover, teachers develop oral exam proposals that, if accepted by an external committee, become the oral exam (DET, 2020). Oral exams are therefore semi-external assessments. Second, municipalities are responsible for developing their own guidelines and for the development and execution of oral exams. Thus, unlike written exams, the content and formats of oral exams are not nationally homogeneous (Regulation of the Education Act, 2006).

### National tests

National tests are low-stakes, digitally administered, standardised tests of basic reading and numeracy skills (Years 5, 8 and 9), and of curriculum-based English reading skills (Years 5 and 8). Their main purpose is to provide information to school owners – i.e. predominantly municipalities – to improve the quality of education. They are compulsory for all students without special education needs or limited communicative competence, and thus map the numeracy, reading and English skills of nearly all students in Norway (DET, 2019; Regulation of the Education Act, 2006).

Reading skills may be associated with differential performance in written and oral assessments (Berninger & Abbott, 2010). Thus, accounting for student scores in national reading tests may improve estimates of concurrent-validity evidence for the interpretation of grades as academic achievement measures.

## Materials and methods

### Sample

Our dataset included information on all Year-10 students in Norway between 2010 and 2018 (*N* = 574,096) and their families. Of these 26,157 students (4.55% of the sample) did not follow a normal progression and thus were considered unrepresentative and excluded from our analytical sample. Specifically 10,307 (1.80%) and 1,601 (0.28%) were students graduating at ages 17 and 15, respectively. Most of these observations likely represent special cases with late and early enrolment. Additionally 10,818 (1.88%) were students graduating below the age of 15 or above the age of 17, 1,781 (0.31%) were students at age 16 who did not graduate in the period of analysis and 1,650 (0.28%) were students at age 16 who graduated in a different year in the period of analysis.

Since our study focuses on the association between grades and written exam results in mathematics, Norwegian and English, we excluded 35,439 students (6.47% of those following the normal progression) without a pair of numeric grades and written exam results in one of those subjects. We also excluded 629 students with more than one pair of grades and written exam results. These observations likely correspond to registration errors or exceptional cases in which students were allowed to take exams in subjects other than the one to which they were randomly assigned. Lastly, we excluded 13 students with only one pair of grades and written exam results but written exams in more than one subject.

The analytical sample (*n* = 511,858) is thus the population of Year-10 students that, between 2010 and 2018, followed a normal progression and had one valid pair of grades and written exam results in mathematics, Norwegian or English.

### Operationalisation

We examined the amount of variance shared by grades and written exams as well as the marginal contributions of possible sources of discrepancy using dominance and multivariate regression analysis.

Dominance analysis (Azen & Budescu, 2003) determines the relative importance of predictors – or sets of predictors – by examining their average marginal contribution to the explained variance of a criterion ($\Delta R^{2}$) in different submodels, which regress the criterion on a linear combination of predictor sets from the base model. This provides meaningful measures of relative importance that are independent of the order in which predictors are included and do not require modelling of the relations between variables (Casillas et al., 2012). Conditional dominance statistics average the marginal contributions of predictor sets in all the submodels of a given order, which refers to the number of predictor sets included in the submodels. A predictor’s conditional dominance statistic measures its within-order average marginal contribution to the explained variance of a criterion. General dominance statistics average the conditional dominance statistics of predictor sets across all orders. A predictor’s general dominance statistic measures its between-order average marginal contribution to the explained variance of the criterion. Finally, standardised general dominance statistics normalise general dominance statistics to add up to 100. Luchman (2021) briefly summarises these techniques. Further technical details can be found in Azen and Budescu (2003).

Following Willingham et al. (2002), we used multivariate regression analysis to correct concurrent predictive models of final grades on written exams by iteratively including indicators of potential content differences, individual differences, situational differences and systematic measurement errors. Accordingly, we specified the following formative model of grades:

$$G_{\mathrm{ijts}}=\beta_{0}E_{\mathrm{ijts}}+\gamma_{C}C_{\mathrm{ij}}+\gamma_{I}I_{i}+T+S+\varepsilon_{\mathrm{ijts}}$$

where i subscripts the individual, j subscripts the subject, t subscripts the cohort and s subscripts the school. Our criterion,$G_{\mathrm{ijts}}$, is Year-10 final grades, and the main predictor,$E_{\mathrm{ijts}}$, is Year-10 written exam results. $C_{\mathrm{ij}}$ is a vector of content difference indicators. $I_{i}$ is a vector of individual difference indicators. Finally, T and S are cohort and school fixed effects accounting for potential situational differences and systematic measurement errors. Since dominance analyses already provide information about the relative importance of predictors, we used unstandardised coefficients in multivariate regression to facilitate the interpretation of dummy variable coefficients in terms of numeric grades. Furthermore, given the size of our sample, all standard errors approach zero; thus, our analysis focused instead on the effect sizes of coefficient estimates.

We also analysed alternative, non-linear specifications, including theory-relevant interactions between indicators of individual and content differences, between written exam results and indicators of situational differences and between written exams and indicators of content differences. The first interaction examines whether indicators of individual differences moderate the association between indicators of content differences and grades predicted by written exams. Willingham et al. (2002) defines individual differences as discrepancies emerging from the interaction between content differences, and background, teacher and student characteristics. However, this interaction is not included in their operationalisation of the theoretical framework. In this specification, large effect sizes of non-interacted indicators of individual differences might better reflect sources of systematic measurement error, such as teachers engaging in problematic grading practices. This interpretation assumes no knowledge domain difference and that our indicator of skill differences captures most of the variation due to the assessment format. The interaction between written exams and situational difference indicators is meant to capture possible discrepancies due to the influence of situational differences through written exam results, e.g. if there were shifts in the scale of exam results across cohorts. Similarly, the interaction between written exam results and our main indicator of content differences is meant to capture whether the influence of content differences on potential discrepancies depends on academic achievement as measured by written exams. We also examined whether different grading standards exist across levels of academic achievement by specifying a model with a quadratic term for written exam results.

Data preparation was carried out in R. Regression analyses and dominance analyses were conducted in Stata.

### Measures

#### Academic achievement

Our predictive formative models assume that grades are determined by academic achievement measures – as measured by written exam results in mathematics, Norwegian and English – and potential sources of discrepancy.

#### Indicators of content differences

##### Indicators of differences in the knowledge domain and non-achievement elements assessed

Since the regulatory framework requires grades and written exam results to measure the same curriculum-based competence, there should not be knowledge domain differences or construct-irrelevant elements affecting the association between grades and written exams at the subject level.

##### National test scores

We used national reading test scores as our main indicator of differences in the skills required to demonstrate academic achievement as measured by grades and written exam results (Berninger & Abbott, 2010). To minimise time bias, we relied on Year-8 reading test scores, which, unlike Year-9 scores, are available for all cohorts. Since national reading test scores were not scaled until 2014, we used standardised-within-cohort raw scores.

##### Oral exam results

A sensitivity analysis included local exam results in oral mathematics, oral English and oral Norwegian as indicators of differences in the oral skills required across assessments. However, results from these models should be interpreted with caution: the inclusion of oral exams substantially reduces the sample size; oral exam results are only semi-external; and the knowledge domain of ‘oral’ subjects does not perfectly overlap with that of ‘written’ subjects.

#### Indicators of individual differences

##### Gender

A dummy variable took a value of 1 if the student was female.

##### Highest parental education

Our data included an ordinal variable taking four possible values for highest parental education: (1) postgraduate education, (2) undergraduate education, (3) upper-secondary education and (4) mandatory education. We derived dummy variables for each level of parental education and used (4) mandatory education as the reference level.

##### Income

We derived deflated aggregate parental net income as our measure of income (NOK 100,000 of 2017). We dealt with negative and extreme values by truncating individual incomes at the 2^nd^ and 98^th^ percentiles of the distribution. To account for the relative financial needs of families, we divided our income measure by equivalised family size (Eurostat, 2021). We assumed that any possible effect of family income on the discrepancy between grades and written exams would be a function of relative changes in income and thus chose to log-transform income. Finally, to ensure the robustness of this variable, we averaged it across the last three years – except for graduates in 2018, for whom we averaged the family income between 2016 and 2017, as those were the only data available in the last three exercises.

##### Parental relationship status

We derived a dummy variable taking a value of 1 for students whose parents cohabited in the same household during graduation year.

##### Foreign background

We derived a dummy variable for students born in a foreign country to foreign-born parents (first-generation migrants) and another for students born in Norway to foreign-born parents (second-generation migrants).

#### Indicators of situational differences

We used cohort and school fixed effects, implemented as dummy variables, to account for systematic differences in the content, context or format of assessments across cohorts and schools.

#### Indicators of measurement error

##### Systematic error

Indicators of individual and situational differences would partly control for the influence of systematic errors associated with student or school characteristics.

##### Unsystematic error

A sensitivity analysis evaluated how error-in-variables regression models (StataCorp, 2021) diverged from multivariate linear regression analyses when using inter-rater intra-class reliability in upper-secondary written exams (Björnsson & Skar, 2021) as a proxy of unsystematic errors in lower-secondary education exams.

## Results

### Descriptive statistics

Table 1 presents the full sample and subsamples of students randomly assigned to written exams in mathematics, Norwegian and English. There were no observable systematic differences in the academic achievement or demographic characteristics of students in different subsamples. Of the students, 91% were native Norwegians. Parental education followed a normal distribution, with only small proportions possessing a mandatory or postgraduate education. The remaining 77% were almost evenly distributed across upper-secondary and tertiary undergraduate degrees. About two-thirds of the students belonged to families in which the parents cohabited in the same household (69%).

**Table 1.** Sample descriptive statistics.

	Full Sample (*N* = 511,858)			Random Draw of Mathematics Exam (*N* = 178,109)			Random Draw of Norwegian Exam (*N* = 160,224)			Random Draw of English Exam (*N* = 173,525)		
Variable	*Missing*	*M*	*SD*	*Missing*	*M*	*SD*	*Missing*	*M*	*SD*	*Missing*	*M*	*SD*
Grades												
Written Mathematics	2,258	3.58	1.22	0	3.60	1.22	1,162	3.56	1.22	1,096	3.57	1.22
Written Norwegian	3,748	3.87	1.01	2,167	3.88	1.01	0	3.85	1.02	1,581	3.87	1.01
Written English	3,218	3.94	1.09	1,848	3.95	1.09	1,370	3.94	1.09	0	3.93	1.09
Written Exams												
Written Mathematics	333,749	3.20	1.25	0	3.20	1.25	160,224	-	-	173,525	-	-
Written Norwegian	351,634	3.46	1.00	178,109	-	-	0	3.46	1.00	173,525	-	-
Written English	338,333	3.74	1.08	178,109	-	-	160,224	-	-	0	3.74	1.08
Oral Exams												
Oral Mathematics	440,598	4.11	1.22	154,464	4.19	1.22	137,680	4.07	1.21	148,454	4.07	1.21
Oral Norwegian	431,388	4.44	1.17	149,593	4.46	1.17	136,668	4.43	1.18	145,127	4.43	1.17
Oral English	431,513	4.46	1.12	148,786	4.46	1.12	134,189	4.45	1.12	148,538	4.48	1.11
Reading Tests^a^	29,997	0.00	1.00	11,211	0.01	1.00	8,597	−0.01	1.00	10,189	0.00	1.00
Gender (Dummy)												
Female	0	0.49	0.50	0	0.49	0.50	0	0.49	0.50	0	0.49	0.50
Highest Parental Education (Dummy)												
Mandatory Education	4,705	0.08	0.27	1,796	0.08	0.27	1,174	0.08	0.27	1,735	0.08	0.27
Upper-secondary	4,705	0.40	0.49	1,796	0.40	0.49	1,174	0.41	0.49	1,735	0.40	0.49
Undergraduate	4,705	0.37	0.48	1,796	0.38	0.48	1,174	0.37	0.48	1,735	0.37	0.48
Postgraduate	4,705	0.15	0.35	1,796	0.15	0.36	1,174	0.14	0.35	1,735	0.15	0.35
Income^b^	1,134	3.58	1.43	449	3.57	1.43	266	3.59	1.43	419	3.58	1.44
Relationship Status (Dummy)												
Together^c^	29,599	0.69	0.46	10,456	0.69	0.46	8,735	0.69	0.46	10,408	0.68	0.47
Foreign Background (Dummy)												
First-Generation Migrant	13,817	0.04	0.20	5,021	0.04	0.20	3,840	0.04	0.19	4,956	0.04	0.20
Second-Generation Migrant	13,817	0.05	0.22	5,021	0.05	0.22	3,840	0.05	0.22	4,956	0.05	0.22

*N* = sample size, *M* = mean, SD = standard deviation.

^a^Normalised within cohorts. ^b^ Equivalised parental net income in NOK 100,000 (2017) averaged across the last three years if available. ^c^ Together = 1 if parents cohabit during the year of graduation.

**Figure 1.**
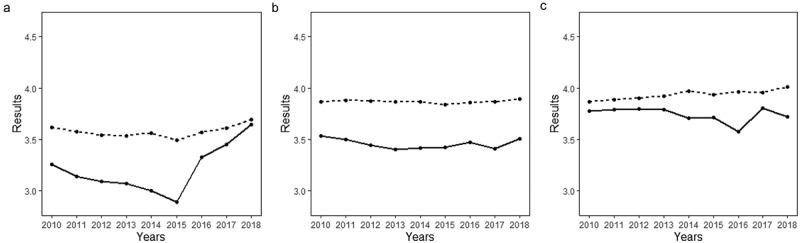
Mean grades and written exam results across years.

*Notes*. Dotted lines represent average grades. Continuous lines represent average written exam results. A) Mathematics grades and written exam results. B) Norwegian grades and written exam results. C) English grades and written exam results.

On average, grades were 0.41 higher than written exam results in Norwegian, 0.38 in mathematics and 0.20 in English (Table A1, in the Appendix). Figure 1 shows how these differences changed over time, particularly in mathematics and English, and how average grades remained relatively stable while average written exam results fluctuated.

Table 2 shows the correlation between grades and written exam results across cohorts. These are rather stable – particularly for assessments in mathematics – and higher than the typical .60 previously reported as the upper range (Ross & Kostuch, 2011).

**Table 2.** Correlations between teacher-assigned grades and written exam results across cohorts.

Subject	2010	2011	2012	2013	2014	2015	2016	2017	2018	Total
Mathematics	.85	.86	.86	.86	.86	.85	.85	.85	.85	.85
Written Norwegian	.64	.65	.66	.66	.68	.69	.70	.70	.70	.68
Written English	.76	.76	.76	.75	.75	.74	.74	.74	.73	.74

*Note.* These are Pearson correlation values between grades and written exams in the same subject, across cohorts and for the full analytic sample.

### Dominance analysis

Table 3 contains the conditional, general and standardised dominance statistics of mathematics grades’ predictors. The standardised dominance statistics indicate that written exam results, on average, led to 66% of the increases in variance explained by predictors of mathematics grades (see ‘Operationalisation’). This is three times higher than our indicator of content differences (22%) (national reading test scores) and nearly eight times higher than our indicators of individual differences (9%). Cohort and school fixed effects – i.e. our indicators of situational differences and systematic errors – led to 1% and 3% average increases, respectively, in the variance explained by predictors of mathematics grades.

**Table 3.** Dominance analysis: mathematics grades.

	Written Exams	Content Differences	Individual Differences	Cohort FE	School FE
Number of Indicators in Submodel	Conditional Dominance				
0	0.71	0.36	0.17	0.00	0.04
1	0.58	0.24	0.09	0.00	0.03
2	0.47	0.14	0.04	0.01	0.02
3	0.39	0.07	0.02	0.01	0.02
4	0.32	0.01	0.00	0.01	0.02
	General Dominance				
	0.50	0.16	0.06	0.01	0.03
	Standardised General Dominance				
	66%	22%	9%	1%	3%

Sample size = 158,214. FE = fixed effects. Conditional dominance statistics equal the average marginal contributions of indicators or sets of indicators (e.g. indicators of individual differences) to the explained variance of submodels with a given number of indicators (i.e. within-order averages). General dominance statistics equal the average conditional dominance statistics across submodels with different numbers of indicators (between-order averages). Standardised general dominance normalises general dominance statistics.

The standardised dominance statistics of Norwegian and English grades’ predictors show the same pattern of decreasing relative importance in written exam results, content differences, individual differences and situational differences and systematic errors observed for mathematics (Tables A2 and A3 in the Appendix). However, the relative importance of potential sources of discrepancy is substantially higher. On average, content differences led to 36% and 33% of the increases in the explained variance of Norwegian and English grades, respectively. Indicators of individual differences led, on average, to 18% and 10% of the increases in the explained variance of Norwegian and English grades, respectively. Finally, the average increase led by cohort fixed effects approached 0% in both subjects, while the increases led by school fixed effects were 5% and 4% in Norwegian and English, respectively.

### Multivariate regression analysis

Table 4 shows the results from robust multivariate regression analyses of the mathematics grades. The bivariate model regressing grades on written exam results for mathematics (Model 1) explains 71% of the variance in grades and suggests that, on average, a 1-point increase in academic achievement as measured by written exams is associated with a linear increase of 0.83 points in grades. This association is, as shown by Model 2, sensitive to the inclusion of content differences – here, in terms of reading test scores – which reduces the coefficient of written exams to 0.74.

**Table 4.** Regression analysis: mathematics grades.

Predictors	Model 1	Model 2	Model 3	Model 4	Model 5	Model 6	Model 7	Model 8	Model 9
Written Exams	0.83***	0.74***	0.73***	0.76***	0.77***	0.74***	0.74***	0.76***	0.81***
Reading Tests^a^		0.16***	0.16***	0.13***	0.13***	0.14***	0.11***	0.10***	0.07***
Gender (Dummy)									
Female			0.06***	0.07***	0.06***	0.06***	0.06***	0.06***	0.06***
Highest Parental Education (Dummy)									
Secondary			0.07***	0.06***	0.05***	0.05***	0.07***	0.07***	0.06***
Undergraduate			0.12***	0.12***	0.11***	0.12***	0.13***	0.13***	0.13***
Postgraduate			0.12***	0.12***	0.16***	0.16***	0.18***	0.18***	0.18***
Income^b^			0.03***	0.04***	0.09***	0.09***	0.09***	0.09***	0.09***
Relationship Status									
Together			0.11***	0.11***	0.11***	0.11***	0.11***	0.11***	0.11***
Foreign Background (Dummy)									
First Generation^c^			0.05***	0.05***	0.09***	0.09***	0.07***	0.07***	0.08***
Second Generation			0.03***	0.04***	0.11***	0.11***	0.11***	0.11***	0.11***
Written Exams^2^						0.01***	0.00***	0.00***	−0.01***
Written Exams × Reading Tests									0.02***
Intercept	0.96***	1.22***	1.03***	0.93***	0.95***	1.00***	0.98***	0.94***	0.86***
Cohort FE				Yes	Yes	Yes	Yes	Yes	Yes
School FE					Yes	Yes	Yes	Yes	Yes
Reading Test × Individual Differences							Yes	Yes	Yes
Written Exam × Cohorts								Yes	Yes
R^2^	0.71	0.72	0.73	0.74	0.76	0.76	0.76	0.76	0.76
N	158,214	158,214	158,214	158,214	158,214	158,214	158,214	158,214	158,214

Coefficients are unstandardised.

^a^Normalised within cohorts. ^b^Natural logarithm of the equivalised parental net income in NOK 100,000 (2017) (averaged across the last three years). ^c^Together = 1 if parents cohabit during the year of graduation.

*p < 0.05, **p < 0.01, ***p < 0.001.

The iterative inclusion of individual differences (Model 3), cohort fixed effects (Model 4) and school fixed effects (Model 5) increased the explained variance of mathematics grades to 76%. Half of this increase (2% points) was driven by the inclusion of school fixed effects.

None of the alternative, non-linear specifications (Models 6–9) increased the total explained variance of the models, but the coefficient estimate of written exam results was somewhat sensitive to the inclusion of a quadratic written exam term (Model 6), the interaction between written exam results and cohort fixed effects (Model 8) and the interaction between written exam results and reading scores (Model 9). In total, the inclusion of sources of discrepancy in the model increased the explained variance of grades by 5%.

Academic achievement has a much more modest predictive power for grades in Norwegian than in mathematics (Table A4 in the Appendix). Model 1 explains only 44% of the variance in Norwegian grades, exceeding the 36% upper limit typically found in previous studies (Ross & Kostuch, 2011) but remaining 27% below the explained variance observed for mathematics grades. In contrast, iteratively introducing content differences (Model 2) and individual differences (Model 3) led to large increases in the explained variance of grades (53% and 57%, respectively).

The coefficient of written exams was very sensitive to the introduction of content and individual differences, dropping from 0.67 to 0.46 in Model 2 and 0.38 in Model 3. Cohort fixed effects (Model 4) and school fixed effects (Model 5) led to relatively modest increases in explained variation (59%) and changes in the effect size of written exam coefficients (0.39). The inclusion of theory-relevant, non-linear independent variables (Models 6–9) had little to no impact on the explained variance of the model. However, the coefficient estimate of written exam results was very sensitive to their inclusion, particularly to the quadratic term of written exams (0.61). The negative coefficient estimate of the quadratic term suggests that teachers are stricter at higher levels of academic achievement.

The pattern of differences in the explained variance of grades and the coefficient estimates of written exam results in English falls between those of mathematics and Norwegian (Table A5 in the Appendix). Model 1 explains 54% of the variance in grades. Other than the inclusion of reading tests (Model 2), which increased the explained variance to 60%, the indicators of potential sources of discrepancy included in Models 3 to 5 made small contributions to the explained variance of English grades. As for mathematics and Norwegian, the coefficient of English written exams was sensitive to the introduction of content differences, dropping from 0.74 to 0.55 in Model 2. The same pattern observed in non-linear predictive models of Norwegian grades was found in predictive models of English grades (Models 6–9).

We used the inter-rater intra-class reliability of upper-secondary education written exams estimated by Björnsson and Skar (2021) to replicate our analyses using error-in-variable regressions (Table A6, in the Appendix). This benchmark analysis produced very large increases in the explained variance of grades and the coefficient estimates of written exams, particularly in Norwegian and English.

The inclusion of oral exam results as additional indicators of content differences in multivariate regression analyses (Table A7, in the Appendix) led to increases of 4% in the explained variance of grades and large relative drops in the effect sizes of written exam results, especially for English.

## Discussion

### Concurrent prediction of grades

We hypothesised that concurrent validity evidence for the interpretation of grades as measures of academic achievement would be stronger in Norway than in contexts in which the constructs assessed by internal and external measures do not overlap sufficiently. This hypothesis was supported by Pearson correlations above .60 for all subjects and across all cohorts. Strong state regulations requiring exams and grades to measure the same underlying construct seem to fulfil this purpose. These results support previous advice against validity arguments that expect a strong correlation between academic achievement measures without careful justification (Brookhart et al., 2016).

Relatively high pairwise correlations between grades and external exams were accompanied by stable average grades, and variations in the average difference between grades and written exam results (see Figure 1). This suggests that changes in average written exam results lead to, according to Willingham et al. (2002)’s nomenclature, ‘trivial’ discrepancies: shifts in the scale of written exams that do not change the order in which students are ranked in a given year. However, because students are randomised into different written exams, scale variations across subjects and – to a lesser extent – cohorts may have important consequences for admission decisions to upper secondary education. While discrepancies that affect the rank order of students are the focus of our study, our results indicate that variations in the scale on which academic achievement measures are reported – e.g. grade inflation – are not only of concern for teacher-assigned grades. Educational researchers and stakeholders should pay as much attention to external assessments and educational assessment system designs as to the ability of teachers to assign grades.

### The role of possible sources of discrepancy between grades and written exams

Our second hypothesis was that state regulations could achieve close construct alignment between internal and external measures but not completely offset potential sources of discrepancy. This is supported by dominance analyses showing that written exams are the most important predictor of grades in Norway but that potential sources of discrepancy account for up to 36% of the between-order average increases in the explained variance by grade predictors. Thus, while strong regulatory frameworks and quality assurance systems can improve the reliability and validity of academic achievement measures, their valid use and interpretation still require accounting for possible sources of discrepancy.

Dominance and multivariate regression analyses showed that content differences are the most important potential source of discrepancies in Norway. Although the regulatory framework focuses on curriculum-based competence goals as the only target construct of grades and written exams, it implicitly recognises that content differences – e.g. in the formats and durations of assessments – may cause discrepancies by specifying that exams measure curriculum-based competences *as demonstrated by students during exams*. Thus, discrepancies due to content differences do not necessarily constitute evidence of invalidity. This implies that, even where the use of concurrent validity evidence is theoretically justified, the optimal correlation between reliable and unbiased measures of academic achievement may not be one.

A potential limitation of our study, however, is uncertainty over whether the best available indicator of content differences in our dataset – reading test scores – indeed serves its purpose. Underlying our strategy is the assumption that reading skills are more important for written assessments than for the set of assessments informing grades. However, the non-interacted (Model 2) and interacted (Model 9) coefficient estimates of reading tests scores were positive and their inclusion (Model 2) led to a large reduction in the coefficient estimates of written exams (Tables 4, A4 and A5). This suggests that part of the explained variance in grades by reading skills and written exams is shared. Reading skills may be a dimension of the construct measured by academic grades and written exams and thus a poor indicator of content differences.

The inclusion of oral exams to account for oral skill differences led to large increases in the explained variance of predicted grades (Table A7). However, the imperfect curriculum overlap between oral and written subjects, the fact that teachers often assess their own students during oral exams and an analytic subsample that may not be representative of the student population in Norway (Falch et al., 2014) advise caution in the interpretation of these results. For example, if oral exams served as a proxy of teacher characteristics instead of oral skills, our results would suggest that, even within a strongly regulated assessment system in an egalitarian society, systematic errors at the teacher level – e.g. strictness or prejudice – threaten the validity of academic achievement measures. From an empirical point of view, this alternative hypothesis is further supported by the sensitivity of individual differences’ coefficient estimates to the inclusion of oral exams.

The interactions between indicators of content differences and individual differences (Model 7) had little influence on the explained variance and remaining coefficient estimates in multivariate regression analyses. If background and student characteristics had large contributions to the explained variance of grades that are independent from content discrepancies, such variables may be better indicators of non-achievement factors influencing teacher grading practices than of individual differences, and thus that problematic grading practices threaten the validity of grades.

Unobserved situational differences and systematic errors captured by school fixed effects accounted for 3%–5% of the increases in the explained variance of grades. This supports previous studies suggesting that school characteristics may influence grades (Wikstrom & Wikstrom, 2005), even in Norway (Galloway et al., 2011), and thus their validity.

Our most complex multivariate regression models explained only between 59% and 76% of the variance in grades. This may be because unreliability in exams heavily attenuates the association between grades and written exams. Our data did not allow us to gauge the reliability of either grades or written exam results, but Table A6 provides a benchmark for the analysis, assuming that selected inter-rater intra-class reliability in upper-secondary written exams (Björnsson & Skar, 2021) constitutes a good proxy for the reliability of lower-secondary written exams. This step increased the variance explained by our base models by 5%–21%. In the most extreme case (Models 4 and 5), our model went from suggesting that increases of 1 point in written exam results were associated with increases of 0.39 in grades to increases of 1.13. Inter-rater reliability estimates in Björnsson and Skar (2021) refer to disagreement before discussion between raters and thus provide a worst-case scenario of reliability. However, our results emphasise the importance of predictor reliability in assessing the validity of academic achievement measures. Using non-standardised and psychometrically tested external assessments of academic achievement may severely underestimate the validity of grades.

Evidence from quasi-concurrent grades and external exam results measuring the same curriculum-based competence in Norway suggest that potential sources of discrepancy contribute to a non-negligible share of the explained variance in grades. However, they do so to a lesser extent than suggested in previous studies, even under the restrictive assumption of perfect external exam reliability. External tests are often implemented with the implicit or explicit argument from policymakers that they provide better ‘objective’ evidence of student achievement with the belief that teachers cannot be trusted to assess students. Our results show that this concern may be unwarranted as grades are better indicators of academic achievement than previously thought. Whether this is dependent on the specific context of Norway, with curriculum-based competence as common target construct and external exams used to homogenise local grading practices, is an open question. Future research from alternative contexts – where external academic achievement assessments and grades target the same construct, and the psychometric properties of external academic achievement assessments are well known – will be able to build on our work and inform us about the generalisability of our results.

### Differences across subjects

The general and standardised dominance statistics of written exams, the model fit of all multivariate regressions and the effect sizes of written exams in predictive models of grades were always higher in mathematics than in Norwegian and English.

This might be explained by our operationalisation of content differences. Reading skills may be an especially relevant part of the curriculum-based competences measured by grades and written exams in English and Norwegian. However, even after their inclusion, the average contribution to the explained variance in grades of indicators of individual differences, for example, was 1% in mathematics and English and 4% in Norwegian (Model 3). Furthermore, the concurrent prediction of Norwegian and English grades by written exams – unlike the concurrent prediction of mathematics grades – was significantly improved with the inclusion of a quadratic term for written exams (Model 6). The negative coefficient estimate of the quadratic term suggests that Norwegian and English grading strictness increases with student academic achievement.

Our results align with previous qualitative findings. For example, Prøitz (2013) found that teachers in conceptually-oriented subjects, such as mathematics, treated their evidence for grading in a more standardised way than in other subjects. In contrast, teachers of Norwegian reported the need for a more norm-referenced approach and acknowledged considering non-achievement elements, such as student effort, attitude and attendance.

## Conclusion

Our results provide empirical support for the argument that construct misalignment is a likely source of discrepancy. Where common operationalisations of academic achievement – e.g. GPA and aggregated test scores – are used and in educational assessment systems with heterogeneous conceptualisations of academic achievement, such as the USA (Brookhart et al., 2016; Willingham et al., 2002; Wittman, 2022), the use of concurrent validity arguments may be inappropriate.

Despite stable and high correlations between grades and written exams, average academic achievement in mathematics, Norwegian and English differed across cohorts and assessments. Consequently, relying solely on these otherwise valid measures for admission decisions might be unfair. Furthermore, student characteristics and school fixed effects, which can threaten the comparability and validity of academic achievement measures, were shown to be important predictors of grades. Therefore, while state regulations and quality assurance systems can enhance the validity of grades, they might not be sufficient to address all potential sources of discrepancies.

We have examined whether the heightened construct overlap between grades and external exams in Norway would yield higher correlations than reported by previous concurrent validation studies (Brookhart et al., 2016; Ross & Kostuch, 2011). Across nearly a decade, the correlation between grades and written exams in mathematics, English and Norwegian never fell below .60. External academic achievement measures in Norway explain 5%–38% more of the variance in grades than we might have expected based on the literature. Furthermore, concerns about the reliability of written exams in lower secondary education suggest that our main results could underestimate the concurrent validity of grades. This has important consequences for far-reaching discussions about the ability of teachers to assign grades: Grades are better measures of academic achievement than previous studies have acknowledged.

## Supplementary Material

Supplemental Material

## Notes

1. There are two written forms of Norwegian: Bokmål and Nynorsk. These are studied in separate subjects, i.e. ‘Norwegian, first choice form, written’ and ‘Norwegian, second-choice form, written’. Lower-secondary education students’ first-choice determines which written form they must use in all their academic work.

2. Most students randomised into written exams in Norwegian, in practice, take two exams, one for each written-form. However, Sami students and some students with foreign backgrounds are exempted from being graded and examined in ‘Norwegian, second-form choice, written’.
