# Supplementary material for: Teacher-assigned grades and external exams: sources of discrepancy
Source: Assess Educ. 2024 Apr 11;31(2):94–115. doi: 10.1080/0969594X.2024.2338764 (PMC11221518; doi:10.1080/0969594X.2024.2338764)
Supplement: Supplemental Material [file CAIE_A_2338764_SM8956.docx]

Table A1: Academic achievement measures: mean and standard deviation across cohorts

| Cohort | Grades | | | Written Exams | | | Oral Exams | | |
| --- | --- | --- | --- | --- | --- | --- | --- | --- | --- |
|  | *n* | *M* | *SD* | *n* | *M* | *SD* | *n* | *M* | *SD* |
|  | Mathematics | | | | | | | | |
| 2010 | 57,099 | 3.62 | 1.19 | 20,090 | 3.26 | 1.19 | 7,145 | 4.11 | 1.20 |
| 2011 | 58,100 | 3.57 | 1.20 | 20,413 | 3.14 | 1.23 | 8,089 | 4.12 | 1.21 |
| 2012 | 56,249 | 3.54 | 1.22 | 21,035 | 3.09 | 1.22 | 7,411 | 4.09 | 1.21 |
| 2013 | 58,064 | 3.53 | 1.22 | 20,215 | 3.07 | 1.28 | 8,274 | 4.12 | 1.23 |
| 2014 | 56,671 | 3.56 | 1.23 | 19,980 | 3.00 | 1.25 | 7,946 | 4.06 | 1.22 |
| 2015 | 57,350 | 3.49 | 1.22 | 19,425 | 2.89 | 1.26 | 8,125 | 4.06 | 1.22 |
| 2016 | 57,031 | 3.57 | 1.24 | 19,743 | 3.33 | 1.22 | 8,311 | 4.11 | 1.22 |
| 2017 | 54,862 | 3.61 | 1.24 | 18,850 | 3.45 | 1.20 | 7,887 | 4.14 | 1.21 |
| 2018 | 54,174 | 3.69 | 1.22 | 18,358 | 3.65 | 1.21 | 8,072 | 4.17 | 1.20 |
| Total | 509,600 | 3.58 | 1.22 | 178,109 | 3.20 | 1.25 | 71,260 | 4.11 | 1.22 |
|  | Norwegian | | | | | | | | |
| 2010 | 56,897 | 3.87 | 0.99 | 17,182 | 3.54 | 0.98 | 8,140 | 4.40 | 1.17 |
| 2011 | 57,960 | 3.88 | 0.99 | 17,829 | 3.50 | 0.98 | 9,194 | 4.42 | 1.16 |
| 2012 | 56,040 | 3.87 | 1.00 | 17,866 | 3.45 | 0.99 | 8,796 | 4.45 | 1.17 |
| 2013 | 57,901 | 3.86 | 1.01 | 18,573 | 3.40 | 0.98 | 9,151 | 4.43 | 1.17 |
| 2014 | 56,506 | 3.86 | 1.01 | 17,468 | 3.42 | 1.02 | 9,289 | 4.41 | 1.17 |
| 2015 | 57,225 | 3.84 | 1.03 | 18,507 | 3.42 | 0.99 | 9,288 | 4.42 | 1.18 |
| 2016 | 56,825 | 3.86 | 1.02 | 18,056 | 3.47 | 1.05 | 9,146 | 4.45 | 1.18 |
| 2017 | 54,684 | 3.87 | 1.03 | 17,621 | 3.41 | 1.00 | 8,724 | 4.47 | 1.18 |
| 2018 | 54,072 | 3.90 | 1.03 | 17,122 | 3.51 | 1.03 | 8,742 | 4.50 | 1.17 |
| Total | 508,110 | 3.87 | 1.01 | 160,224 | 3.46 | 1.00 | 80,470 | 4.44 | 1.17 |
|  | English | | | | | | | | |
| 2010 | 56,844 | 3.87 | 1.08 | 20,028 | 3.78 | 1.07 | 8,394 | 4.37 | 1.10 |
| 2011 | 57,887 | 3.89 | 1.09 | 20,117 | 3.79 | 1.09 | 9,634 | 4.40 | 1.12 |
| 2012 | 56,048 | 3.90 | 1.10 | 17,553 | 3.80 | 1.06 | 8,418 | 4.42 | 1.12 |
| 2013 | 57,987 | 3.93 | 1.09 | 19,500 | 3.79 | 1.07 | 9,095 | 4.47 | 1.11 |
| 2014 | 56,559 | 3.97 | 1.09 | 19,418 | 3.70 | 1.09 | 9,199 | 4.46 | 1.13 |
| 2015 | 57,339 | 3.94 | 1.10 | 19,689 | 3.72 | 1.07 | 9,248 | 4.46 | 1.12 |
| 2016 | 57,013 | 3.96 | 1.09 | 19,495 | 3.58 | 1.10 | 8,995 | 4.50 | 1.12 |
| 2017 | 54,836 | 3.96 | 1.08 | 18,710 | 3.81 | 1.07 | 8,680 | 4.52 | 1.11 |
| 2018 | 54,127 | 4.01 | 1.08 | 19,015 | 3.72 | 1.09 | 8,682 | 4.56 | 1.12 |
| Total | 508,640 | 3.94 | 1.09 | 173,525 | 3.74 | 1.08 | 80,345 | 4.46 | 1.12 |

*Note*. N = sample size, M = mean, SD = standard deviation

Table A2: Dominance analysis: written Norwegian grades

|  | Written Exams | Content Differences | Individual Differences | Cohort FE | School FE |
| --- | --- | --- | --- | --- | --- |
| Number of Indicators in Submodel | Conditional Dominance | | | | |
| 0 | 0.44 | 0.39 | 0.23 | 0.00 | 0.04 |
| 1 | 0.32 | 0.28 | 0.14 | 0.00 | 0.03 |
| 2 | 0.22 | 0.19 | 0.08 | 0.00 | 0.03 |
| 3 | 0.14 | 0.12 | 0.05 | 0.00 | 0.03 |
| 4 | 0.09 | 0.08 | 0.03 | 0.00 | 0.03 |
|  | General Dominance | | | | |
|  | 0.24 | 0.21 | 0.11 | 0.00 | 0.03 |
|  | Standardised General Dominance | | | | |
|  | 41 % | 36 % | 18 % | 0 % | 5 % |

*Note*. Sample size = 143,815. FE = fixed effects. Conditional dominance statistics equal the average marginal contributions of indicators or sets of indicators (e.g. indicators of individual differences) to the explained variance of submodels with a given number of indicators (i.e. within-order averages). General dominance statistics equal the average conditional dominance statistics across submodels with different numbers of indicators (between-order averages). Standardised general dominance normalises general dominance statistics.

Table A3: Dominance analysis: written English grades

|  | Written Exams | Content Differences | Individual Differences | Cohort FE | School FE |
| --- | --- | --- | --- | --- | --- |
| Number of Indicators in Submodel | Conditional Dominance | | | | |
| 0 | 0.54 | 0.42 | 0.15 | 0.00 | 0.04 |
| 1 | 0.42 | 0.29 | 0.08 | 0.00 | 0.03 |
| 2 | 0.31 | 0.19 | 0.04 | 0.00 | 0.02 |
| 3 | 0.23 | 0.11 | 0.02 | 0.00 | 0.02 |
| 4 | 0.16 | 0.05 | 0.01 | 0.00 | 0.02 |
|  | General Dominance | | | | |
|  | 0.33 | 0.21 | 0.06 | 0.00 | 0.03 |
|  | Standardised General Dominance | | | | |
|  | 52 % | 33 % | 10 % | 0 % | 4 % |

*Note*. Sample size = 154,465. FE = fixed effects. Conditional dominance statistics equal the average marginal contributions of indicators or sets of indicators (e.g. indicators of individual differences) to the explained variance of submodels with a given number of indicators (i.e. within-order averages). General dominance statistics equal the average conditional dominance statistics across submodels with different numbers of indicators (between-order averages). Standardised general dominance normalises general dominance statistics.

Table A4: Regression analysis: written Norwegian grades

| Predictors | Model 1 | Model 2 | Model 3 | Model 4 | Model 5 | Model 6 | Model 7 | Model 8 | Model 9 |
| --- | --- | --- | --- | --- | --- | --- | --- | --- | --- |
| Written Exams | 0.67*** | 0.46*** | 0.38*** | 0.38*** | 0.39*** | 0.61*** | 0.62*** | 0.59*** | 0.65*** |
| Reading Tests ^a^ |  | 0.37*** | 0.35*** | 0.35*** | 0.36*** | 0.35*** | 0.35*** | 0.36*** | 0.31*** |
| Gender (Dummy) |  |  |  |  |  |  |  |  |  |
| Female |  |  | 0.34*** | 0.34*** | 0.34*** | 0.33*** | 0.33*** | 0.33*** | 0.33*** |
| Highest Parental Education (Dummy) |  |  |  |  |  |  |  |  |  |
| Secondary |  |  | 0.10*** | 0.10*** | 0.09*** | 0.08*** | 0.10*** | 0.10*** | 0.10*** |
| Undergraduate |  |  | 0.23*** | 0.23*** | 0.23*** | 0.22*** | 0.24*** | 0.24*** | 0.23*** |
| Postgraduate |  |  | 0.28*** | 0.28*** | 0.31*** | 0.31*** | 0.32*** | 0.33*** | 0.32*** |
| Income ^b^ |  |  | 0.05*** | 0.05*** | 0.09*** | 0.09*** | 0.09*** | 0.09*** | 0.09*** |
| Relationship Status |  |  |  |  |  |  |  |  |  |
| Together |  |  | 0.12*** | 0.12*** | 0.12*** | 0.12*** | 0.12*** | 0.12*** | 0.12*** |
| Foreign Background (Dummy) |  |  |  |  |  |  |  |  |  |
| First Generation ^c^ |  |  | -0.01 | -0.01 | 0.02 | 0.02 | -0.01 | -0.01 | -0.01 |
| Second Generation |  |  | 0.02* | 0.02** | 0.06*** | 0.05*** | 0.05*** | 0.05*** | 0.05*** |
| Written Exams^2^ |  |  |  |  |  | -0.03*** | -0.03*** | -0.03*** | -0.04*** |
| Written Exams $\times$ Reading Tests |  |  |  |  |  |  |  |  | 0.02*** |
| Intercept | 1.55*** | 2.29*** | 2.07*** | 2.05*** | 2.14*** | 1.79*** | 1.75*** | 1.86*** | 1.76*** |
| Cohort FE |  |  |  | Yes | Yes | Yes | Yes | Yes | Yes |
| School FE |  |  |  |  | Yes | Yes | Yes | Yes | Yes |
| Reading Test $\times$ Individual Differences |  |  |  |  |  |  | Yes | Yes | Yes |
| Written Exam $\times$ Cohorts |  |  |  |  |  |  |  | Yes | Yes |
| R^2^ | 0.44 | 0.53 | 0.57 | 0.57 | 0.59 | 0.59 | 0.59 | 0.59 | 0.60 |
| N | 143,815 | 143,815 | 143,815 | 143,815 | 143,815 | 143,815 | 143,815 | 143,815 | 143,815 |

*Notes*. Coefficients are unstandardised. ^a^ Normalised within cohorts. ^b^ Natural logarithm of the equivalised parental net income in NOK 100,000 (2017) (averaged across the last three years). ^c^ Together = 1 if parents cohabit during the year of graduation.

* p<0.05, ** p<0.01, *** p<0.001

Table A5: Regression analysis: written English grades

| Predictors | Model 1 | Model 2 | Model 3 | Model 4 | Model 5 | Model 6 | Model 7 | Model 8 | Model 9 |
| --- | --- | --- | --- | --- | --- | --- | --- | --- | --- |
| Written Exams | 0.74*** | 0.55*** | 0.52*** | 0.53*** | 0.54*** | 0.74*** | 0.74*** | 0.77*** | 0.79*** |
| Reading Tests ^a^ |  | 0.33*** | 0.32*** | 0.31*** | 0.31*** | 0.31*** | 0.30*** | 0.30*** | 0.28*** |
| Gender (Dummy) |  |  |  |  |  |  |  |  |  |
| Female |  |  | 0.16*** | 0.16*** | 0.16*** | 0.15*** | 0.15*** | 0.15*** | 0.15*** |
| Highest Parental Education (Dummy) |  |  |  |  |  |  |  |  |  |
| Secondary |  |  | 0.07*** | 0.06*** | 0.07*** | 0.06*** | 0.07*** | 0.07*** | 0.07*** |
| Undergraduate |  |  | 0.16*** | 0.15*** | 0.16*** | 0.16*** | 0.17*** | 0.17*** | 0.16*** |
| Postgraduate |  |  | 0.21*** | 0.20*** | 0.24*** | 0.24*** | 0.25*** | 0.25*** | 0.25*** |
| Income ^b^ |  |  | 0.05*** | 0.03*** | 0.06*** | 0.06*** | 0.06*** | 0.06*** | 0.06*** |
| Relationship Status |  |  |  |  |  |  |  |  |  |
| Together |  |  | 0.06*** | 0.06*** | 0.06*** | 0.06*** | 0.06*** | 0.06*** | 0.06*** |
| Foreign Background (Dummy) |  |  |  |  |  |  |  |  |  |
| First Generation ^c^ |  |  | 0.15*** | 0.13*** | 0.15*** | 0.15*** | 0.15*** | 0.15*** | 0.16*** |
| Second Generation |  |  | 0.15*** | 0.13*** | 0.16*** | 0.16*** | 0.15*** | 0.15*** | 0.15*** |
| Written Exams^2^ |  |  |  |  |  | -0.03*** | -0.03*** | -0.03*** | -0.03*** |
| Written Exams $\times$ Reading Tests |  |  |  |  |  |  |  |  | 0.01** |
| Intercept | 1.18*** | 1.90*** | 1.67*** | 1.60*** | 1.58*** | 1.22*** | 1.21*** | 1.13*** | 1.09*** |
| Cohort FE |  |  |  | Yes | Yes | Yes | Yes | Yes | Yes |
| School FE |  |  |  |  | Yes | Yes | Yes | Yes | Yes |
| Reading Test $\times$ Individual Differences |  |  |  |  |  |  | Yes | Yes | Yes |
| Written Exam $\times$ Cohorts |  |  |  |  |  |  |  | Yes | Yes |
| R^2^ | 0.54 | 0.60 | 0.61 | 0.61 | 0.64 | 0.64 | 0.64 | 0.64 | 0.64 |
| N | 154,465 | 154,465 | 154,465 | 154,465 | 154,465 | 154,465 | 154,465 | 154,465 | 154,465 |

*Notes*. Coefficients are unstandardised. ^a^ Normalised within cohorts. ^b^ Natural logarithm of the equivalised parental net income in NOK 100,000 (2017) (averaged across the last three years). ^c^ Together = 1 if parents cohabit during the year of graduation.

* p<0.05, ** p<0.01, *** p<0.001

Table A6: Reliability-corrected predictive models

| Predictor | Mathematics | | | Norwegian | | | English | |
| --- | --- | --- | --- | --- | --- | --- | --- | --- |
|  | (1) | (2) | (3) | (4) | (5) | (6) | (7) | (8) |
| Written Exams | 0.77*** | 0.93*** | 0.89*** | 0.39*** | 1.13*** | 1.02*** | 0.54*** | 1.24*** |
| Reading Tests | 0.13*** | 0.03*** | 0.05*** | 0.36*** | -0.01* | 0.04*** | 0.31*** | -0.12*** |
| Foreign Background (Dummy) |  |  |  |  |  |  |  |  |
| First Generation | 0.09*** | 0.06*** | 0.07*** | 0.02 | 0.02 | 0.02 | 0.15*** | -0.05*** |
| Second Generation | 0.11*** | 0.07*** | 0.08*** | 0.06*** | 0.02 | 0.02* | 0.16*** | -0.05*** |
| Gender (Dummy) |  |  |  |  |  |  |  |  |
| Female | 0.06*** | 0.07*** | 0.07*** | 0.34*** | -0.00 | 0.05*** | 0.16*** | 0.03*** |
| Relationship Status (Dummy) |  |  |  |  |  |  |  |  |
| Together | 0.11*** | 0.07*** | 0.08*** | 0.12*** | 0.03*** | 0.05*** | 0.06*** | 0.03*** |
| Income | 0.09*** | 0.05*** | 0.06*** | 0.09*** | 0.02* | 0.03*** | 0.06*** | 0.00 |
| Highest Parental Education (Dummy) |  |  |  |  |  |  |  |  |
| Upper-Secondary | 0.05*** | 0.02* | 0.02*** | 0.09*** | 0.04*** | 0.05*** | 0.07*** | 0.02* |
| Undergraduate | 0.11*** | 0.04*** | 0.06*** | 0.23*** | 0.07*** | 0.09*** | 0.16*** | 0.02* |
| Postgraduate | 0.16*** | 0.05*** | 0.08*** | 0.31*** | 0.08*** | 0.11*** | 0.24*** | 0.01 |
| Intercept | 0.95*** | 0.58*** | 0.67*** | 2.14*** | -0.01 | 0.29** | 1.58*** | -0.86*** |
| Cohort FE | Yes | Yes | Yes | Yes | Yes | Yes | Yes | Yes |
| Schools FE | Yes | Yes | Yes | Yes | Yes | Yes | Yes | Yes |
| Reliability (Benchmark) | - | .91 | .93 | - | .62 | .64 | - | .68 |
| R^2^ | 0.76 | 0.83 | 0.81 | 0.59 | 0.76 | 0.73 | 0.64 | 0.85 |
| N | 158,214 | 158,214 | 158,214 | 143,815 | 143,815 | 143,815 | 154,465 | 154,465 |

*Notes*. Coefficients are unstandardised. Reliability benchmarks (selected from Björnsson & Skar, 2021): (2) MAT1015, Mathematics 2P; (3) MAT1011, Mathematics 1P; (6) NOR1211, First-choice form of Norwegian, written; (7) NOR1212, Second form of Norwegian, written; (8) ENG1002, English.

^a^ Normalised within cohorts. ^b^ Natural logarithm of the equivalised parental net income in NOK 100,000 (2017) (averaged across the last three years). ^c^ Together = 1 if parents cohabit during the year of graduation.

* p<0.05, ** p<0.01, *** p<0.001

Table A7: Regression analyses with and without oral exams

| Predictors | Mathematics | | Norwegian | | English | |
| --- | --- | --- | --- | --- | --- | --- |
|  | (1) | (2) | (3) | (4) | (5) | (6) |
| Written Exams | 0.77*** | 0.57*** | 0.39*** | 0.30*** | 0.54*** | 0.41*** |
| Oral Exams |  | 0.30*** |  | 0.24*** |  | 0.27*** |
| Reading Tests | 0.14*** | 0.10*** | 0.36*** | 0.27*** | 0.30*** | 0.23*** |
| Foreign Background (Dummy) |  |  |  |  |  |  |
| First Generation | 0.08** | 0.06** | 0.06* | -0.00 | 0.13*** | 0.09*** |
| Second Generation | 0.11*** | 0.08*** | 0.07** | 0.03 | 0.15*** | 0.10*** |
| Gender(Dummy) |  |  |  |  |  |  |
| Female | 0.08*** | 0.03*** | 0.33*** | 0.27*** | 0.18*** | 0.17*** |
| Relationship Status (Dummy) |  |  |  |  |  |  |
| Together | 0.11*** | 0.08*** | 0.12*** | 0.07*** | 0.07*** | 0.05*** |
| Income | 0.08*** | 0.05*** | 0.07*** | 0.04* | 0.05*** | 0.02 |
| Highest Parental Education |  |  |  |  |  |  |
| Secondary | 0.04* | 0.03 | 0.09*** | 0.06** | 0.07*** | 0.06** |
| Undergraduate | 0.10*** | 0.05** | 0.24*** | 0.17*** | 0.16*** | 0.11*** |
| Postgraduate | 0.14*** | 0.08*** | 0.32*** | 0.22*** | 0.22*** | 0.16*** |
| Intercept | 0.75** | 0.21 | 2.03*** | 1.31*** | 1.69*** | 1.02*** |
| CohortFE | Yes | Yes | Yes | Yes | Yes | Yes |
| Schools FE | Yes | Yes | Yes | Yes | Yes | Yes |
| R2 | 0.76 | 0.80 | 0.62 | 0.66 | 0.65 | 0.69 |
| N | 20,880 | 20,880 | 21,164 | 21,164 | 22,114 | 22,114 |

*Notes*. Coefficients are unstandardised.

^a^ Normalised within cohorts. ^b^ Natural logarithm of the equivalised parental net income in NOK 100,000 (2017) (averaged across the last three years). ^c^ Together = 1 if parents cohabit during the year of graduation.

* p<0.05, ** p<0.01, *** p<0.001

Table A8. Correlation table

|  |  | (1) | (2) | (3) | (4) | (5) | (6) | (7) | (8) | (9) | (10) | (11) | (12) | (13) | (14) | (15) | (16) | (17) | (18) | (19) |
| --- | --- | --- | --- | --- | --- | --- | --- | --- | --- | --- | --- | --- | --- | --- | --- | --- | --- | --- | --- | --- |
| Written Mathematics ^a^ | (1) | 1.00 |  |  |  |  |  |  |  |  |  |  |  |  |  |  |  |  |  |  |
| Written Norwegian ^a^ | (2) | 0.64 | 1.00 |  |  |  |  |  |  |  |  |  |  |  |  |  |  |  |  |  |
| Written English ^a^ | (3) | 0.59 | 0.72 | 1.00 |  |  |  |  |  |  |  |  |  |  |  |  |  |  |  |  |
| Written Mathematics ^b^ | (4) | 0.85 | 0.60 | 0.58 | 1.00 |  |  |  |  |  |  |  |  |  |  |  |  |  |  |  |
| Written Norwegian ^b^ | (5) | 0.54 | 0.68 | 0.62 | 0.00 | 1.00 |  |  |  |  |  |  |  |  |  |  |  |  |  |  |
| Written English ^b^ | (6) | 0.53 | 0.63 | 0.74 | 0.00 | 0.00 | 1.00 |  |  |  |  |  |  |  |  |  |  |  |  |  |
| Oral Mathematics ^c^ | (7) | 0.78 | 0.58 | 0.54 | 0.75 | 0.50 | 0.48 | 1.00 |  |  |  |  |  |  |  |  |  |  |  |  |
| Oral Norwegian ^c^ | (8) | 0.59 | 0.66 | 0.59 | 0.57 | 0.58 | 0.54 | 0.82 | 1.00 |  |  |  |  |  |  |  |  |  |  |  |
| Oral English ^c^ | (9) | 0.47 | 0.54 | 0.67 | 0.47 | 0.47 | 0.62 | 0.62 | 0.43 | 1.00 |  |  |  |  |  |  |  |  |  |  |
| Reading Tests ^d^ | (10) | 0.60 | 0.63 | 0.65 | 0.61 | 0.58 | 0.62 | 0.53 | 0.54 | 0.52 | 1.00 |  |  |  |  |  |  |  |  |  |
| Female ^†^ | (11) | 0.08 | 0.32 | 0.19 | 0.06 | 0.29 | 0.16 | 0.11 | 0.24 | 0.10 | 0.13 | 1.00 |  |  |  |  |  |  |  |  |
| Mandatory Education ^e†^ | (12) | -0.20 | -0.18 | -0.17 | -0.20 | -0.16 | -0.16 | -0.18 | -0.16 | -0.14 | -0.19 | 0.00 | 1.00 |  |  |  |  |  |  |  |
| Upper-secondary ^e†^ | (13) | -0.23 | -0.20 | -0.20 | -0.24 | -0.18 | -0.19 | -0.21 | -0.20 | -0.19 | -0.20 | 0.00 | -0.24 | 1.00 |  |  |  |  |  |  |
| Undergraduate ^e†^ | (14) | 0.15 | 0.15 | 0.14 | 0.15 | 0.13 | 0.12 | 0.14 | 0.15 | 0.13 | 0.14 | 0.00 | -0.22 | -0.63 | 1.00 |  |  |  |  |  |
| Postgraduate ^e†^ | (15) | 0.25 | 0.21 | 0.22 | 0.27 | 0.20 | 0.21 | 0.22 | 0.20 | 0.20 | 0.22 | 0.00 | -0.12 | -0.34 | -0.32 | 1.00 |  |  |  |  |
| Income ^f^ | (16) | 0.20 | 0.19 | 0.18 | 0.22 | 0.18 | 0.17 | 0.18 | 0.17 | 0.16 | 0.21 | 0.00 | -0.21 | -0.17 | 0.09 | 0.27 | 1.00 |  |  |  |
| Parents Cohabit ^†^ | (17) | 0.19 | 0.13 | 0.10 | 0.17 | 0.10 | 0.08 | 0.16 | 0.14 | 0.09 | 0.09 | 0.00 | -0.06 | -0.08 | 0.05 | 0.09 | -0.24 | 1.00 |  |  |
| First-Generation Migrant ^g†^ | (18) | -0.07 | -0.11 | -0.07 | -0.08 | -0.09 | -0.07 | -0.06 | -0.06 | -0.05 | -0.13 | 0.00 | 0.16 | -0.04 | -0.05 | 0.01 | -0.19 | 0.08 | 1.00 |  |
| Second-Generation Migrant ^g†^ | (19) | -0.04 | -0.06 | -0.01 | -0.03 | -0.06 | -0.02 | -0.03 | -0.03 | -0.01 | -0.10 | 0.00 | 0.17 | -0.01 | -0.06 | -0.03 | -0.16 | 0.06 | -0.05 | 1.00 |

*Notes*. Pearson correlation among all included variables (excluded cohort and school indicators) for all pairwise complete cases in our full analytical sample (N = 511,858).

^a^ Teacher-assigned grades. ^b^ Written exam results. ^c^ Oral exam results. ^d^ Normalised within cohorts. ^e^ Dummy indicators of highest parental education. ^f^ Equivalised parental net income in NOK 100,000 (2017) averaged across the last three years if available. ^g^ Dummy indicators of foreign background. † Dummy variable.
